# Supplementary material for: Distinctive tasks of different cyanobacteria and associated bacteria in carbon as well as nitrogen fixation and cycling in a late stage Baltic Sea bloom
Source: PLoS One. 2019 Dec 12;14(12):e0223294. doi: 10.1371/journal.pone.0223294 (PMC6907833; doi:10.1371/journal.pone.0223294)
Supplement: S1 Table — Abiotic variables at the day of sampling at station TransA. (DOCX) [file pone.0223294.s001.docx]

**S1 Table:**

Sampling conditions at station TransA.

| Date | Temp (°C) | Salinity (PSU) | Oxygen (ml/l) | NO_3_ (µmol/l) | PO_4_ (µmol/l) |
| --- | --- | --- | --- | --- | --- |
| 13.08.2015 | 16.5 | 6.2 | 7.4 | <0.02 | <0.02 |

Abiotic variables at the day of sampling at station TransA.
