# Supplementary material for: Distinctive tasks of different cyanobacteria and associated bacteria in carbon as well as nitrogen fixation and cycling in a late stage Baltic Sea bloom
Source: PLoS One. 2019 Dec 12;14(12):e0223294. doi: 10.1371/journal.pone.0223294 (PMC6907833; doi:10.1371/journal.pone.0223294)
Supplement: S2 Table — A: Numbers of analysed cells per species/group as well as total analysed areas (which may contain different species/bacterial groups) for each incubation time. B: Numbers of analysed associated bacterial cells with the respective cyanobacterial host species for each incubation time. (DOCX) [file pone.0223294.s002.docx]

**S2 Table:**

**Regions of interest (ROIs) analysed.**

A: Numbers of analysed cells per species/group as well as total analysed areas (which may contain different species/bacterial groups) for each incubation time. B: Numbers of analysed associated bacterial cells with the respective cyanobacterial host species for each incubation time.

A:

| Incubation time/  host species | 10 min | 30 min | 60 min | 6 h | 24 h |
| --- | --- | --- | --- | --- | --- |
| Aphanizomenon sp. | 31 | 35 | 33 | 34 | 13 |
| Dolichospermum sp. | 47 | 27 | 57 | 23 | 22 |
| Nodularia sp. | 31 | 6 | 28 | 24 | 28 |
| Pseudanabaena sp. | 23 | 16 | 11 | 12 | 17 |
| Alphaproteo | 9 | 2 | 7 | 10 | 7 |
| Bacteroidetes/Cytophaga | 15 | 5 | 15 | 5 | 13 |
| Total analysed areas | 36 | 29 | 34 | 24 | 27 |

B:

| Associated bacteria | Incubation time/  host species | 10 min | 30 min | 60 min | 6 h | 24 h |
| --- | --- | --- | --- | --- | --- | --- |
| Alphaproteo | Aphanizomenon sp. | 1 | 2 | 1 | 5 | - |
|  | Dolichospermum sp. | 3 | - | 2 | - | - |
|  | Nodularia sp. | 5 | - | 4 | 5 | 7 |
|  | Pseudanabaena sp. | - | - | - | - | - |
| Bacteroidetes/ | Aphanizomenon sp. | 5 | 1 | 7 | 2 | 3 |
| Cytophaga | Dolichospermum sp. | 3 | 2 | 3 | 1 | 1 |
|  | Nodularia sp. | 6 | 2 | 5 | 2 | 8 |
|  | Pseudanabaena sp. | 1 | - | - | - | 1 |
