# Supplementary material for: Distinctive tasks of different cyanobacteria and associated bacteria in carbon as well as nitrogen fixation and cycling in a late stage Baltic Sea bloom
Source: PLoS One. 2019 Dec 12;14(12):e0223294. doi: 10.1371/journal.pone.0223294 (PMC6907833; doi:10.1371/journal.pone.0223294)
Supplement: S4 Table — ANOVAs and Post-Hoc analyses for differences in 13C and 15N uptake for the different species over time as well as differences in 13C and 15N uptake by the associated bacteria caused by different hosts. (DOCX) [file pone.0223294.s004.docx]

**S4 Table: Statistics for ^13^C and ^15^N uptake.**

ANOVAs for the uptake of ^13^C and ^15^N for the different species over time

| Species | ^13^C uptake | ^15^N uptake |
| --- | --- | --- |
| Aphanizomenon sp. | F = 5.602  p = 0.000 | F = 8.321  p = 0.000 |
| Dolichospermum sp. | F = 21.43  p = 0.000 | F = 10.97  p = 0.000 |
| Nodularia sp. | F = 1.372  p = 0.248 | F = 3.974  p = 0.005 |
| Pseudanabaena sp. | F = 18.16  p = 0.000 | F = 65.43  p = 0.000 |
| Alphaproteobacteria | F = 2.024  p = 0.117 | F = 4.87  p = 0.003 |
| Cytophaga/Bacteroidetes bacteria | F = 6.579  p = 0.000 | F = 9.811  p = 0.000 |

HSD Posthoc tests for the uptake of ^13^C and ^15^N for the different species over time

| species | Aphanizomenon sp. | | Dolichospermum sp. | | Nodularia sp. | | Pseudanabaena sp. | | Alphaproteobacteria | | Cytophaga/Bacteroidetes bacteria | |
| --- | --- | --- | --- | --- | --- | --- | --- | --- | --- | --- | --- | --- |
| time | ^13^C | ^15^N | ^13^C | ^15^N | ^13^C | ^15^N | ^13^C | ^15^N | ^13^C | ^15^N | ^13^C | ^15^N |
| 10 min | B | B | B | B | A | A | B | C | A | B | B | B |
| 30 min | B | B | B | B | A | AB | B | C | A | AB | B | B |
| 60 min | B | B | B | B | A | AB | B | C | A | AB | B | B |
| 6 h | A | A | B | A | A | AB | B | B | A | A | AB | AB |
| 24 h | B | B | A | A | A | B | A | A | A | A | A | A |

ANOVAs for differences in ^13^C and ^15^N uptake by bacteria associated to different hosts

| time | ^13^C uptake | ^15^N uptake |
| --- | --- | --- |
| 10 min | F = 0.839  p = 0.557 | F = 3.15  p = 0.029 |
| 30 min | F = 0.849  p = 0.552 | F = 1.249  p = 0.43 |
| 60 min | F = 10.15  p = 0.001 | F = 9.689  p = 0.000 |
| 6 h | F = 2.47  p = 0.112 | F = 6.117  p = 0.009 |
| 24 h | F = 0.932  p = 0.472 | F = 1.926  p = 0.158 |

HSD Posthoc tests for differences in ^13^C and ^15^N uptake by the bacteria associated to different hosts. No associated bacteria to specific hosts are indicated with -

|  | time | 10 min | | 30 min | | 60 min | | 6 h | | 24 h | |
| --- | --- | --- | --- | --- | --- | --- | --- | --- | --- | --- | --- |
| associated bacterial group | Host species | ^13^C | ^15^N | ^13^C | ^15^N | ^13^C | ^15^N | ^13^C | ^15^N | ^13^C | ^15^N |
| Alphaproteo | Aphanizomenon sp. | A | B | A | A | AB | AB | A | B | - | - |
| Alphaproteo | Dolichospermum sp. | A | AB | - | - | ABC | B | - | - | - | - |
| Alphaproteo | Nodularia sp. | A | B | - | - | C | B | A | A | A | A |
| Alphaproteo | Pseudanabaena sp. | - | - | - | - | - | - | - | - | - | - |
| Cytophaga | Aphanizomenon sp. | A | AB | A | A | BC | B | A | B | A | A |
| Cytophaga | Dolichospermum sp. | A | A | A | A | AB | B | A | B | A | A |
| Cytophaga | Nodularia sp. | A | B | A | A | A | A | A | AB | A | A |
| Cytophaga | Pseudanabaena sp. | A | AB | - | - | - | - | - | - | A | A |
