# Supplementary material for: Distinctive tasks of different cyanobacteria and associated bacteria in carbon as well as nitrogen fixation and cycling in a late stage Baltic Sea bloom
Source: PLoS One. 2019 Dec 12;14(12):e0223294. doi: 10.1371/journal.pone.0223294 (PMC6907833; doi:10.1371/journal.pone.0223294)
Supplement: Attachment — Submitted filename: Response to reviewer.docx [file pone.0223294.s007.docx]

Response to reviewer

| In this manuscript Eigmann et al. used Nano-SIMS to evaluate carbon and nitrogen fixation within a late-stage cyanobacterial bloom in the Baltic Sea. Surprisingly, they found considerable nitrogen fixation within heterotrophic bacteria and Pseudanabaena, and little to no fixation among heterocystous species.  Although the study is limited to a single sample (?) – time-series observations during bloom formation and collapse would have been very interesting – I found the results and analysis compelling. There are numerous small errors that need to be corrected before publication. | We thank the reviewer for his profound comments and criticisms. The time series (10 min, 30 min, 1h, 6 h, 24 h) was set up in separate bottles, but the reviewer is right that they all originated from one location and one time point of the bloom. |
| --- | --- |
| Citation styles are mixed, and the bibliography is incomplete | We apologize for these mistakes. Please see the revised version for corrections. |
| Line 32 – suggest “by NanoSims” | Changed as suggested. |
| Line 37 – “but were also” is confusing, aren’t these the same thing? | The associated bacteria recycled nitrogen that was fixed by other organisms, but were also indicative of nitrogen fixation. Changed to:“… dominated the subsequent nitrogen remineralization with uptake rates up to 1.2 ± 1.93 fmol N h-1 cell -1, but were also indicative for fixation of di-nitrogen.” |
| Line 41 – “mass” is an odd term to use here, suggest bloom | Changed as suggested. |
| Line 43-45 – sentence starting “The onset of” is nonsensical, please revise | Changed to: “Start of blooms…“ |
| Line 46 – at not “with” | Changed as suggested. |
| Line 48 – up to not “for” | Changed as suggested. |
| Line 51 – the mention of eukaryotic phytoplankton is a distraction, as they are not the subject of this study | Changed to: „Cyanobacteria live in close associations….“ |
| Line 54 – here and elsewhere, “therewith” is not a word | Chaged to „thereby“. |
| Line 65 – suggest incapable in place of “not capable” | Changed as suggested. |
| Line 66 – remove “and hence depend on dissolved nitrogen sources” as this is self-evident | Removed as suggested. |
| Line 67 – I’m not an expert on these strains but a quick search for “Pseudanabaena nitrogen fixation” returned lots of hits with evidence of nitrogen fixation. | It is known for Pseudanabaena that it carries genes for nitrogen fixation. It was never shown, however, to perform nitrogen fixation in the Baltic Sea, despite several studies were performed on it. This might be related to the stage of the bloom, i.e. we sampled a late stage bloom, and other studies concentrated on active blooms. |
| Line 70 – estuary not “estuarine” | We apologize for this mistake. Changed as suggested. |
| Line 76 – delete “technique” | Deleted as suggested. |
| Line 84 – I’m not familiar with light traps, but as described it doesn’t make much sense; the zooplankton are removed but how does this result in concentration of cyanobacteria? What was the in situ chlorophyll concentration? | Changed to: „Positive phototactic zooplankton was removed by means of a light trap and bloom samples were concentrated until a cyanobacterial chl. a concentration…”  The original cyanobacterial chl. a concentration was ca. 4 µg l-1. |
| Line 106 – standard methods call for a 0.2 um filter for bacterial collection. Do you think you adequately captured the heterotrophic population? | No, we for sure did not capture the full heterotrophic population/diversity. This was, however, also not our goal. We were only interested in the directly to the cyanobacteria attached fraction of heterotrophic bacteria. After several pre-studies we decided to use 3-µm porewidth filter for this.  We added: „…pore width (we only aimed at the directly attached heterotrophic bacterial fraction) polycarbonate…” |
| Line 176 – “specific cell metabolism” requires some further explanation | Changed to: „…of heterocysts in Aphanizomenon sp., Dolichospermum sp., and Nodularia sp. were avoided due to rapid transfer of fixed nitrogen.” |
| Line 194 – where not “whereof” | Corrected as suggested. |
| Line 212 – I’m not following the logic of this statement; do you mean absolute numbers of cyanobacteria were not available? | For cyanobacteria absolute numbers were available. Because of this, for cyanobacteria we could calculate uptake rates per volume per time. For the associated bacteria on the other hand, one can not calculate absolute numbers (they may be covered by organic material, attached to the backside of the host aso…). Because of this, we could only calculate uptake rates per cell for the associated bacteria.  For better understanding we changed this part to:  „For cyanobacteria gross uptake rates could be calculated per volume and time (absolute numbers were known). For the associated bacteria uptake rates were calculated per cell and time, because no absolute numbers of associated bacteria were existent.” |
| Line 213 – the meaning of “2x1” is not clear | Changed to: „…with 2 x1 (length x width) µm…” |
| Line 214 – suggest “carbon content of … ratio of…” | Changed as suggested. |
| Line 222 – cite R before R Studio | Citation changed as suggested. |
| Line 233 – cite vegan | Cited as suggested. |
| Table 1 – given the different units this table is a little hard to follow. I suggest using an asterisk or other symbol to identify those lines that correspond to heterotrophs. | Changed as suggested. Please see changes in the track changes version. |
| Line 295-296 – these two sentences seem to be in opposition… | Changed to: „For all time points, significant differences of 15N incorporation between the species/groups occurred (Fig. 6). After 30 min Pseudanabaena sp. (which reveals the highest 15N incorporation), and…” |
| Line 323 – “Anova” should be capitalized | Changed as suggested. |
| Line 341 – confirm that these a r, not r2 | Yes, these are R values. R values are proxies for the degree of differences between different groups analysed by ANOSIM. |
| Line 352 – use time not “the time” | Changed as suggested. |
| Line 409 – P-value should not be hyphenated | Changed to: „…be adapted to this situation where phosphorus supply by degrading blooms may…” |
| Line 444 – Comparable not “comparably” | Changed as suggested. |
| Line 447 – do you mean one order of magnitude rather than one dimension? | Changed to: „…i.e. approximately one order of magnitude above…” |
| Line 451 – suggest consistent with not “congenial” | Changed as suggested. |
